# Transcriptome-wide association study reveals two genes that influence mismatch negativity

**DOI:** 10.1016/j.celrep.2021.108868

**Published:** 2021-03-16

**Authors:** Anjali Bhat, Haritz Irizar, Johan Hilge Thygesen, Karoline Kuchenbaecker, Oliver Pain, Rick A. Adams, Eirini Zartaloudi, Jasmine Harju-Seppänen, Isabelle Austin-Zimmerman, Baihan Wang, Rebecca Muir, Ann Summerfelt, Xiaoming Michael Du, Heather Bruce, Patricio O’Donnell, Deepak P. Srivastava, Karl Friston, L. Elliot Hong, Mei-Hua Hall, Elvira Bramon

**Affiliations:** 1 Division of Psychiatry, University College London, London, UK; 2 Social, Genetic, and Developmental Psychiatry Centre, Institute of Psychiatry, Psychology, and Neuroscience, King’s College London, London, UK; 3 Wellcome Centre for Human Neuroimaging, University College London, London, UK; 4 Institute of Cognitive Neuroscience, University College London, London, UK; 5 UCL Genetics Institute, University College London, London, UK; 6 Maryland Psychiatric Research Center, Department of Psychiatry, University of Maryland, Baltimore, MD, USA; 7 Department of Psychiatry, Harvard Medical School, Boston, MA, USA; 8 Psychosis Neurobiology Laboratory, McLean Hospital, Belmont, MA, USA; 9 Institute of Psychiatry, Psychology, and Neuroscience, King’s College London, London, UK; 10 Camden and Islington NHS Foundation Trust, London, UK; 11 Department of Basic and Clinical Neuroscience, Institute of Psychiatry, Psychology, and Neuroscience, King’s College London, London, UK; 12 MRC Centre for Neurodevelopmental Disorders, King’s College London, London, UK; 13 Takeda Pharmaceuticals, Cambridge, MA, USA; 14 Department of Clinical, Educational and Health Psychology, University College London, London, UK

**Keywords:** mismatch negativity, psychosis, endophenotype, schizophrenia, transcriptome-wide association study, prediction error, Bayesian brain, gene expression, neurodevelopment, MMN

## Abstract

Mismatch negativity (MMN) is a differential electrophysiological response measuring cortical adaptability to unpredictable stimuli. MMN is consistently attenuated in patients with psychosis. However, the genetics of MMN are uncharted, limiting the validation of MMN as a psychosis endophenotype. Here, we perform a transcriptome-wide association study of 728 individuals, which reveals 2 genes (*FAM89A* and *ENGASE*) whose expression in cortical tissues is associated with MMN. Enrichment analyses of neurodevelopmental expression signatures show that genes associated with MMN tend to be overexpressed in the frontal cortex during prenatal development but are significantly downregulated in adulthood. Endophenotype ranking value calculations comparing MMN and three other candidate psychosis endophenotypes (lateral ventricular volume and two auditory-verbal learning measures) find MMN to be considerably superior. These results yield promising insights into sensory processing in the cortex and endorse the notion of MMN as a psychosis endophenotype.

## Highlights

• Expression of *FAM89A* and *ENGASE* is associated with reduced mismatch negativity (MMN)
• Genes regulating neurotransmitter levels increase in MMN transcriptome-wide association
• Genes influencing MMN are underexpressed in adult brain cortex
• MMN ranks above verbal memory and ventricular volume as psychosis endophenotype

## Graphical abstract

**Figure:**
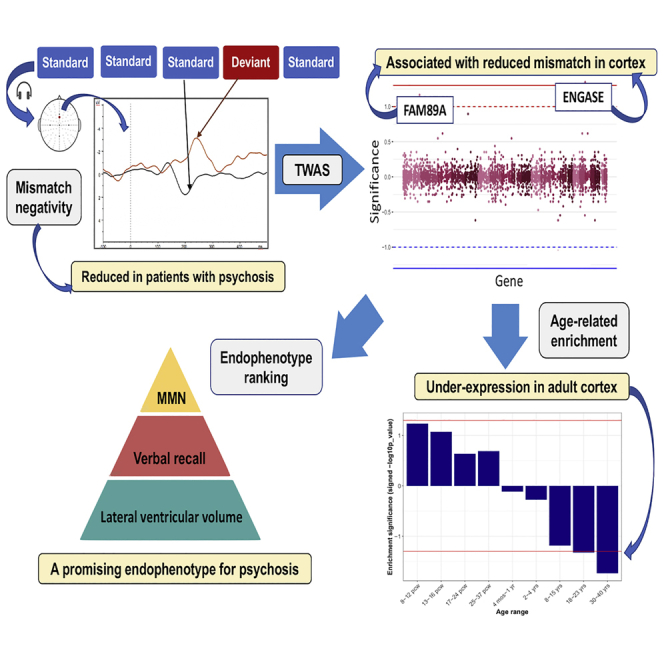

## Teaser

Bhat et al. identify two genes, *FAM89A* and *ENGASE*, whose expression in cortical tissue is negatively associated with mismatch negativity (MMN), an electrophysiological measure of cortical responses to unexpected sensory stimuli. They find enrichment of neurotransmission-regulating genes in these associations and endorse MMN as an endophenotype for psychosis.

## Introduction

Mismatch negativity (MMN) is an event-related potential that measures the cortical response to occasional “oddball” stimuli in an otherwise repetitive series (Naatanen, 1992; Näätänen et al., 1978). MMN is interpreted as a “prediction error signal,” the brain’s response to sensory information that deviates from its prior “beliefs” (Friston, 2005; Garrido et al., 2009). This does not refer to propositional beliefs (participants in the MMN paradigm are instructed not to pay attention to stimuli presented), but rather an unconscious predictive processing that frames the brain as a statistical model of its environment which generates predictions about sensations, compares it to actual sensory input, and updates itself to minimize discrepancies (Helmholtz, 1962; Knill and Pouget, 2004). This updating depends upon the relative precision of prior beliefs and sensory evidence. Here, “precision” is a measure of certainty and physiologically represents post-synaptic gain (excitability) of neurons reporting prediction errors (Parr and Friston, 2019). The lower the precision of sensory data (e.g., the more muffled a sound) or the higher the precision of prior beliefs (e.g., the more times the sound has been heard), the less readily these neurons fire action potentials, much as a scientist would ascribe reliability to experimental findings and weigh them against an existing body of literature (Parr and Friston, 2019). The MMN oddball paradigm is widely used because of its profundity, replicability, and simplicity as a measure of how a brain adapts to a changing environment. The most common MMN paradigm involves presenting participants with “standard” and “deviant” tones (Erickson et al., 2016). The deviance can be in a variety of domains, including intensity and frequency or duration, as long as it departs from an established pattern (Kathmann et al., 1999). The MMN waveform is quantified as the difference between the event-related potentials elicited by the standard and deviant stimuli (Baldeweg and Hirsch, 2015).

Psychosis is a highly heritable mental disorder characterized by hallucinations, delusions, and cognitive deficits (American Psychiatric Association, 2013; Bergen et al., 2012; Anttila et al., 2018; Hilker et al., 2018; Thygesen et al., 2020). It has recently been conceptualized as a disorder of aberrant precision—the precision weighting of sensory stimuli is skewed (Limongi et al., 2018). This literature is very recent, so the specific mechanisms of this aberrant precision are not well understood, but it has been suggested that prior beliefs may be “overweighted” in hallucinations (Benrimoh et al., 2019; Powers et al., 2017) and “underweighted” in the case of the MMN (Adams et al., 2013; Sterzer et al., 2018) relative to new sensory information. This is supported by the fact that patients with psychosis consistently show significantly smaller MMN than do healthy controls (Erickson et al., 2016; Näätänen et al., 2012; Shelley et al., 1991). Moreover, MMN is attenuated in patients before illness onset (Bramon et al., 2004; Hong et al., 2012) and is predictive of the transition to psychosis in high-risk patients (Bodatsch et al., 2011; Erickson et al., 2016). MMN also progresses with the disorder. First-episode psychosis patients show less attenuated MMN than chronic patients (Erickson et al., 2016; Haigh et al., 2017). MMN is therefore considered a strong candidate endophenotype for psychosis.

Endophenotypes are biomarkers of structure or function that characterize an illness and indicate genetic liability (Bramon et al., 2005; Gottesman and Gould, 2003). Psychotic disorders such as schizophrenia and bipolar disorder with psychotic symptoms are heterogeneous and highly polygenic—>100 genetic loci have been associated with schizophrenia and >30 with bipolar disorder with psychotic symptoms (Pardiñas et al., 2018; Bramon et al., 2014; Stahl et al., 2019). The mechanisms by which these genetic variants affect the disease pathway remain unclear. Studying the genetics of a well-defined and objectively quantifiable endophenotype such as MMN has strong potential to yield key insights into the biological mechanisms of psychosis development. However, an important criterion for a trait being a useful endophenotype is a substantial overlap in genetic architecture with the disease itself (Calafato and Bramon, 2019; Iacono et al., 2017). The likelihood of such an overlap has been indicated by research that shows attenuated mismatch in unaffected relatives of people with psychosis (Bramon et al., 2014), but targeted genetic association methods have yet to be applied to MMN to substantiate this phenomenon.

Transcriptome-wide association studies (TWASs), like genome-wide association studies (GWASs), are a useful hypothesis-free method of studying how genetic variation influences a trait. Both GWAS and TWAS have been central to the study of psychosis, having identified 145 genetic loci and 175 genes, respectively, that are reliably associated with schizophrenia (Gusev et al., 2018; Pardiñas et al., 2018; Bramon et al., 2014). While GWASs evaluate variation at the single-nucleotide polymorphism (SNP) level, TWASs evaluate variation at a gene level (Gamazon et al., 2015, 2019; Gusev et al., 2018; Huckins et al., 2019). This is valuable for phenotypes such as MMN that are laborious to obtain and rarely collected in combination with genetic data, as the lower multiple-testing burden of gene-level associations allows TWAS to be well powered with much smaller sample sizes. Analyzing gene expression also allows more direct inference of biological mechanisms. It is often difficult to deduce which biological pathways are implicated by GWAS-significant SNPs due to linkage disequilibrium and the poorly understood dynamics of non-coding regions of the genome (Gusev et al., 2018). The TWAS approach makes it possible to infer gene expression in a discovery dataset without having to collect tissue expression data. Specifically, TWASs evaluate the association between individual differences in genetically regulated gene expression and an outcome of interest. Expression levels are inferred based on a preexisting reference dataset that contains both genotype and tissue expression data (e.g., the Genotype-Tissue Expression [GTEx] Project database, which we use in the present study). For these reasons, we considered a TWAS to be the most appropriate method of exploring the genetics of MMN.

The tissues we have selected from GTEx are the Brodmann area 9 (BA9) region of the frontal cortex, as well as the whole cortex; these were chosen for their relevance to the phenotype. Previous functional magnetic resonance imaging (fMRI) studies have shown that auditory MMN localizes to the inferior frontal gyrus (IFG) and superior temporal gyrus (STG) (Doeller et al., 2003; Opitz et al., 2002). These gene expression data are derived from post-mortem tissue samples (which would have had to be physically sliced and then pulped and centrifuged to extract RNA) and do not benefit from the task-based localization afforded by neuroimaging methods such as fMRI, but a broader level of localization is sufficient for the purposes of this study, as there is a very high likelihood of shared genetic signals between adjacent tissues (Ip et al., 2018). Although the two tissues overlap, we have chosen to analyze both for two reasons. First, the STG is best accounted for by the “whole cortex” tissue, as tissue samples localized to the STG are not available in the GTEx database, or, to our knowledge, any other open-source databases. Second, there is a larger (and not entirely overlapping) set of genes available from GTEx for the whole cortex than there is for the frontal cortex.

Studying the genetic architecture of processes that underlie MMN may elucidate biological and neurodevelopmental mechanisms that underlie sensory processing as well as psychosis. In this study, we aim to identify genes whose expression in cortical tissues are associated with MMN, assess their relevance over the lifespan, and evaluate MMN as a psychosis endophenotype.

## Results

### Demographics

After all quality control procedures, a total of 728 participants (302 with psychosis and 426 healthy controls) with both genetic and MMN data were available for analysis (unaffected relatives from the London sample were treated as healthy controls in the present study; see Method details). There was no significant difference in age between control (mean = 40.25 years; SD = 15.28 years) and patient (mean = 38.70 years; SD = 12.92 years) groups across the whole sample (n = 728, t = 1.431, p = 0.153). There was a significantly smaller proportion of female participants among patients (31.79%) compared to controls (58.45%) across the whole sample (n = 728, χ^2^ = 49.325, p = 2.169e^−12^). A description of the sample can be seen in Table 1.

**Table 1:** Demographics and clinical characteristics of the sample after genetic quality control

	Maryland			Harvard			London			
	Patient	Control	Overall	Patient	Control	Overall	Patient	Relative	Control	Overall
N	164	239	403	54	17	71	84	82	88	254
Females, %	28	56.5	44.9	38.9	58.8	43.7	34.5	59.8	62.6	52.4
Age, y^a^	36.1 ± 13.5	38.7 ± 16.1	37.6 ± 15.1	43.8 ± 11.3	36.9 ± 15.9	42.1 ± 12.8	40.5 ± 11.7	46.7 ± 13.8	39.2 ± 12.5	42.0 ± 13.0
Age range^a^	11–63	9–80	9–80	21–66	21–63	21–66	18–65	17–73	18–62	17–73

a Mean ± SD.

### MMN amplitude is attenuated in patients with psychosis

Table 2 shows the average MMN (with SD) for each dataset included in our sample, as well as for patients, relatives (for Psychosis Endophenotypes International Consortium [PEIC]), and controls within each dataset. Please see Figures S1 and S2 for MMN waveform plots for the Maryland (n = 403) and Harvard (n = 71) samples. MMN plots from the London sample (n = 254) have been published previously in Bramon et al. (2004) and Ranlund et al. (2016).

**Table 2:** Mean mismatch negativity amplitude at Fz (μV) in each of the datasets by group

Sample	Patients	Controls	Relatives	Whole dataset	Decrease, %^a^
Maryland^b^	−1.09 ± 1.43	−1.59 ± 1.78	–	−1.39 ± 1.66	31.7
Harvard^b^	−1.10 ± 2.20	−2.18 ± 2.27	–	−1.36 ± 2.25	49.4
PEIC^b^	−2.52 ± 1.24	−3.15 ± 1.59	−3.27 ± 1.56	−2.98 ± 1.5	20.1

Amplitude shown as mean ± SD (in μV).

a These values are unadjusted for covariates.

b Percentage reduction in patients compared to unaffected subjects (average of relatives and controls, for PEIC).

Age-, gender-, and lab-adjusted linear regressions revealed a significantly attenuated MMN Fz peak amplitude in psychosis patients in the whole sample (n = 728, effect size = 0.70 μV, 95% CI = 0.45–0.94 μV, p = 3.5e^−8^), as well as in each of the 2 largest datasets (Maryland: n = 403, coef. = 0.48 μV, 95% CI = 0.14–0.82 μV, p = 6e^−3^; London: n = 254, coef. = 0.66 μV, 95% CI = 0.23–1.11 μV, p = 3e^−3^). We did not see any difference in MMN in psychosis patients in the smallest sample (Harvard; n = 71, coef. = −0.01 μV; 95% CI = −1.59 to 1.57 μV, p = 0.99), most likely due to the limited statistical power of this sample (a sample of 71 is too small to yield meaningful genetic association results on its own). However, in the combined sample of 728 participants, the group comparisons are consistent with the literature, with significantly reduced MMN amplitude in patients with psychosis (Table S3).

Within the London sample, a linear regression, which included age, gender, and testing laboratory as covariates, showed no significant difference in MMN amplitude at the Fz electrode between unaffected relatives of patients with psychosis and healthy controls. Unaffected relatives appeared to have slightly (insignificantly) enhanced MMN compared to controls (effect size = −0.297, SE = 0.234, p = 0.205, 95% CI = −0.76 to 0.16).

To assess the effect of stimulus duration on MMN, we compared our cohorts that used shorter stimuli (London and Harvard) with the cohort that used longer auditory stimuli (Maryland) in their MMN paradigm, by linear regression with age, gender, and clinical group as covariates. In the Maryland group (n = 403, mean = −1.39 μV, SD = 1.66 μV), MMN was smaller than the shorter stimuli groups (n = 325, mean = −2.63 μV, SD = 1.82 μV). The model explained 17.47% of the variance and was a significant predictor of MMN amplitude: F(5,722) = 30.56, p < 2.2 × 10^−16^; stimulus length contributed significantly to this difference (effect size = −1.16 μV, p = 2.63 × 10^−16^). The latency of MMN at Fz in the Maryland group (mean = 186.6 ms, SD = 29.63 ms) was later than the shorter stimuli groups (n = 166, mean = 132.19, SD = 45.95); stimulus length contributed significantly to this difference (effect size = −44.35, p < 2 × 10^−16^).

### Increased family with sequence similarity 89 member A (*FAM89A*) and endo-β-*N*-acetylglucosaminidase (*ENGASE*) expression is associated with attenuated MMN

**Figure 1:**
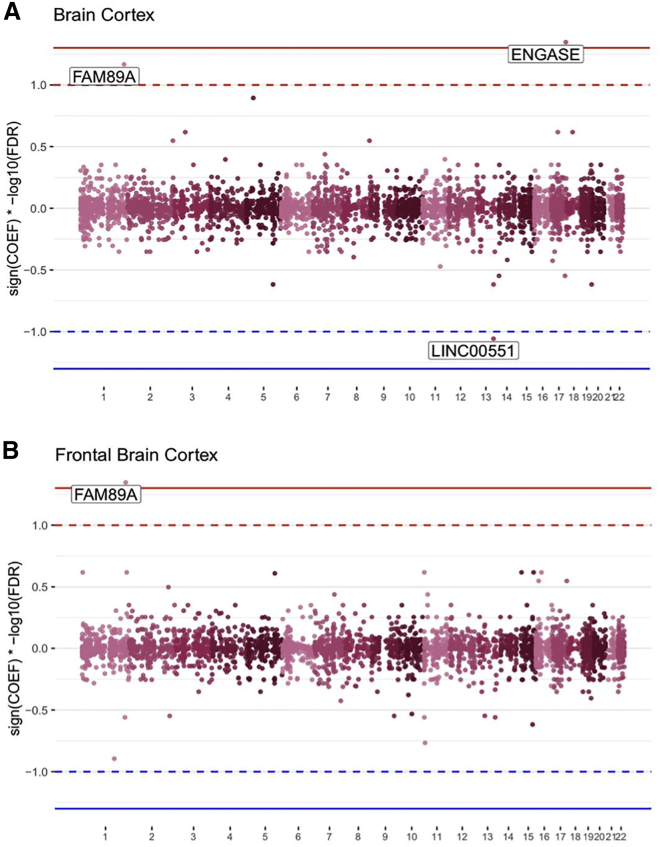
Transcriptome-wide association of 4,329 and 3,604 genes in the cortex and frontal cortex, respectively, shows *FAM89A* and *ENGASE* to be significantly associated with MMN The Manhattan plots show, by tissue, the significance (−log_10_ FDR) of all of the genes in the TWAS of MMN, multiplied by the sign of the coefficient to show the direction of the effect [(sign(coefficient)]. (A) Predicted expression of *ENGASE* in the whole cortex is significantly positively associated with MMN peak amplitude at the FDR < 0.05 threshold indicated by the solid line. Genes within the dotted line show a (non-significant) association with MMN within a threshold of FDR < 0.1. (B) Predicted expression of *FAM89A* in the frontal cortex is significantly (FDR < 0.05) positively associated with MMN peak amplitude.

**Figure 2:**
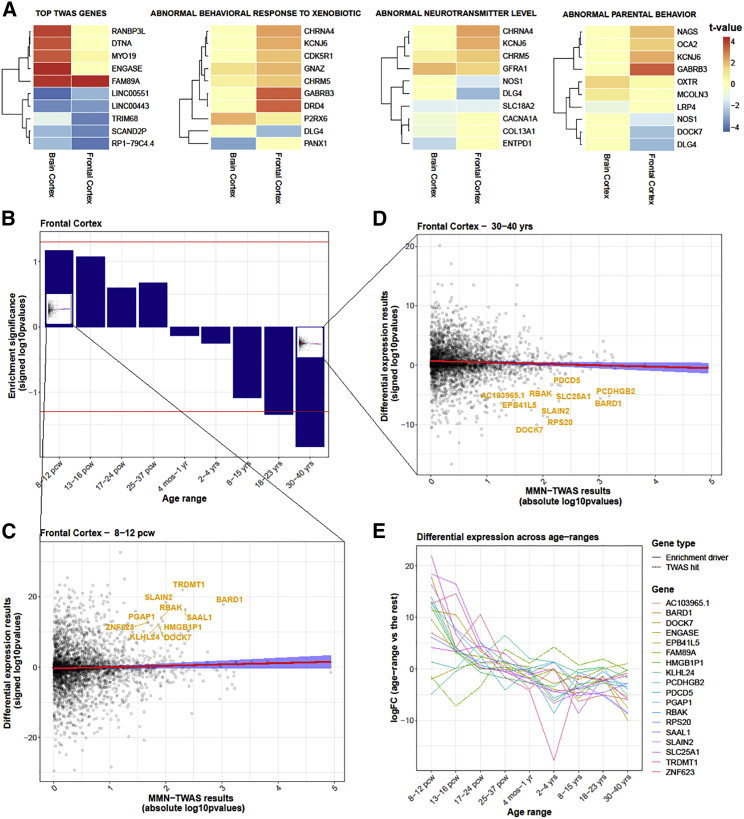
Gene set and neurodevelopmental stage enrichment analyses of MMN TWAS results show enrichment of genes controlling neurotransmitter level and downregulation of MMN-associated genes in adulthood (A) The first heatmap shows association strengths (t statistics) and directions of association (red, positive; blue, negative) of the top 10 MMN TWAS genes. The other heatmaps correspond to each of the 3 gene sets enriched for MMN and the constituent genes primarily driving these gene set-MMN associations. (B) Expression of MMN-related genes across neurodevelopmental stages. Each bar of the bar plot represents an age range and the p value of an underlying association analysis, which assessed whether the genes more strongly associated to MMN in the TWAS were significantly up- or downregulated in the frontal cortex within that age range. The last 2 bars reach the threshold of p < 0.05, showing a significant downregulation of MMN-related genes in older age groups. (C) The regression analysis represented by the first bar of the bar plot, which tests the association between (absolute value of) p values of genes examined in the MMN TWAS and the (signed −log_10_) p values of genes assessed in a differential expression analysis between each age range and all of the others. The slope shows a slight positive relationship between MMN-related genes and the gene expression profile of the 8- to 12-week post-conception neurodevelopmental stage. (D) The regression analysis represented by the last bar of the bar plot, showing a negative relationship between MMN-related genes from the TWAS and the gene expression profile of the 30–40 years neurodevelopmental stage. (E) Differential expression across the 9 age range categories of the 16 genes most responsible for driving the trend in the downregulation of MMN-related genes.

In the TWAS of MMN peak amplitude, at the Benjamini-Hochberg-corrected significance threshold (false discovery rate [FDR] = 0.05), there are 2 genes that were significantly positively associated with MMN (Figure 1): *ENGASE* in whole cortex (effect size = 1.09; p = 1.06e−5; FDR = 0.045; 95% CI = 0.60–1.58) and *FAM89A* in the frontal cortex (effect size = 0.82; p = 1.1e−5; FDR = 0.045; 95% CI = 0.46–1.19). Multiple test correction was performed to account for all of the genes tested across both tissues. A heatmap showing the strength and direction of association for the top 10 genes in the TWAS can be found in Figure 2A. For the entire table of TWAS results from all genes included in the analysis, please see Table S2. The MMN peak is the negative component of the waveform obtained by subtracting the response to the standard stimulus from the response to the deviant stimulus. This means here that higher expression of *FAM89A* or *ENGASE* results in attenuated MMN amplitudes. For the frontal cortex, there were 26 SNPs in the PrediXcan gene model for *FAM89A* (*R*^*2*^ [i.e., prediction accuracy] *=* 0.2798, p = 2.01 × 10^−8^). For the whole cortex, there were 40 SNPs in the PrediXcan model for *FAM89A* (*R*^*2*^ *=* 0.3471, p *=* 1.86 × 10^−12^) and 23 SNPs in the gene model for *ENGASE* (*R*^*2*^ = 0.0361, p = 0.0426).

### Genes controlling neurotransmitter levels are enriched in MMN associations

In our MMN-TWAS results for the frontal cortex, one gene set (abnormal neurotransmitter level) is significantly enriched (overrepresented) at the FDR < 0.05 threshold (Table 3). We did not find any significantly enriched gene sets in the whole cortex.

**Table 3:** One gene set enriched for MMN at FDR < 0.05 and two (non-significant) at FDR < 0.1

Gene set	Genes in set	Genes tested	β	SE	t stat	p	FDR
Abnormal neurotransmitter level	69	11	0.6579	0.187	3.5213	2.15 × 10^−4^	0.045
Abnormal parental behavior	204	20	0.4624	0.140	3.3027	4.79 × 10^−4^	0.050
Abnormal behavioral response to xenobiotic	79	12	0.2945	0.097	3.0354	1.20 × 10^−3^	0.084

FDR, false discovery rate; MMN, mismatch negativity; SE, standard error (β).

### Genes that influence MMN are underexpressed in adulthood

In our neurodevelopmental signature enrichment analysis, genes more strongly associated with MMN in the TWAS for frontal cortex are significantly underexpressed in the adult age categories of 18–23 years (effect size = −.0086, p = 0.0473, SE = 0.0043) and 30–40 years (effect size = −0.0074, p = 0.0185, SE = 0.0032). Although not significant, the prenatal stages show a relative upregulation of higher p value TWAS genes (Figure 2B). A rank-based identification of the top 10 genes driving the association between the TWAS results and gene expression within the earliest (8–12 weeks post-conception) and latest (and 30–40 years) categories (Figures 2C and 2D, respectively) reveals 4 genes (*BARD1, RBAK, SLAIN2*, and *DOCK7*, all ranked within the top 30 genes in the MMN TWAS results) that are strongly overexpressed in the early prenatal stage and strongly downregulated in adulthood. The top 10 genes driving this result also individually show a marked overexpression in the earliest prenatal stage and a gradual decrease in expression over neurodevelopment, although *ENGASE* and *FAM89A* do not follow this pattern (Figure 2E). The significant downregulation in adulthood was not seen in the whole cortex, but nominal prenatal overexpression can also be observed here (see Figure S3).

### MMN ranks higher than working memory and ventricular volume as a psychosis endophenotype

**Figure 3:**
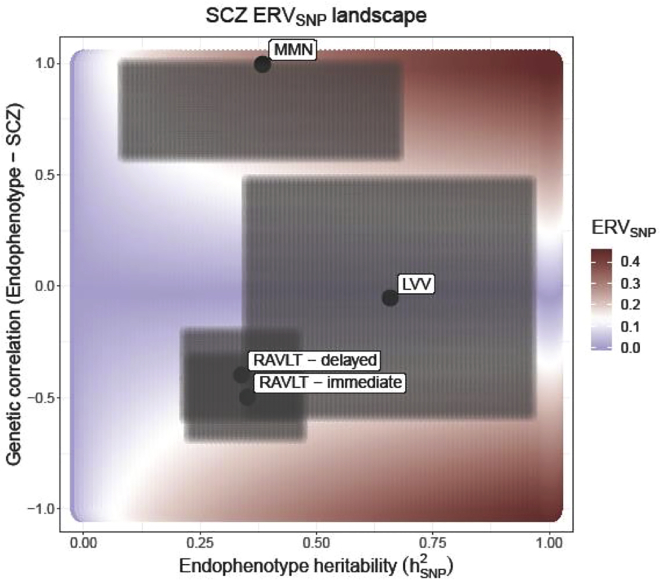
MMN ranks higher than working memory and ventricular volume in comparison of endophenotype ranking values (ERV_SNP_) of schizophrenia candidate endophenotypes The graph shows a comparison of SNP-based endophenotype ranking values (ERV_SNP_) of MMN compared to ERV_SNP_s of Rey Auditory Verbal Learning Task (RAVLT) immediate recall, RAVLT delayed recall, and brain lateral ventricular volume (LVV). The color scale indicates the ERV strength: a good endophenotype would sit in darker red or darker blue areas, indicating that it has a high degree of pleiotropy with the disease and is strongly heritable itself. ERV here has a maximum value of 0.447, given a SNP-based heritability (*h*^2^_SNP_) for schizophrenia of 0.2002 (Pardiñas et al., 2018). The shaded confidence areas in gray indicate the SEs of the endophenotype heritability estimates (*h*^2^_SNP_) along the x axis and of the genetic overlap between the endophenotype and schizophrenia along the y axis. The ERV is a standardized genetic covariance and does not have units.

To estimate the utility of MMN to understand more about the genetics of psychosis, we calculated the SNP-based endophenotype ranking value (ERV_SNP_, a 0–1 scale value representing genetic overlap between phenotype and illness; Glahn et al., 2012) of MMN and 3 comparator phenotypes that have previously been associated with psychosis risk (Thygesen et al., 2020). The results (Figure 3) show that the ERV_SNP_ for MMN (0.28) is substantially higher (there is no overlap between the lower bound of the confidence area and the upper bounds thereof for the other endophenotypes) than those of lateral ventricular volume (LVV; 0.02), Rey Auditory Verbal Learning Task (RAVLT) delayed (0.10), and RAVLT immediate (0.13). ERV is a standardized covariance, so it does not have units; it is calculated based on heritability and genetic correlation estimates for each phenotype. These estimates have wide confidence intervals in our analysis (Table S7), so they must be interpreted cautiously.

## Discussion

Our TWAS of mismatch negativity MMN revealed two genes whose expression is significantly positively associated with MMN: *FAM89A* (frontal cortex) and *ENGASE* (whole cortex). This means that increased expression of these genes relates to attenuated MMN amplitudes. Both genes are protein coding, but relatively little is known about their functions. *FAM89A* encodes a protein that contributes to cytoskeletal organization, modulation of protein synthesis, and neurite outgrowth (Gürol et al., 2015). It is highly expressed in placental tissue and interacts with the biogenetic protein *UBX2NB*, which is highly expressed in the fetal brain (https://www.string-db.org), suggesting a role for *FAM89A* in prenatal neurodevelopment. Functionally, *FAM89A* appears to be primarily involved in the immune response. It differentiates between viral and bacterial infections (Gómez-Carballa et al., 2019), is implicated in glial tumors (Pan et al., 2019), and is downregulated on exposure to interleukins 10 and 13 (Alevy et al., 2012; Trandem et al., 2011).

ENGASE acts as a cytosolic enzyme that breaks down oligosaccharides and is involved in the degradation of asparagine-linked N-glycans (Shi and Trimmer, 1999; Suzuki et al., 2002). Asparagine-linked N-glycosylation patterns influence the unique functional properties of potassium channels in the mammalian brain (Shi and Trimmer, 1999). The signaling of sensory precision is thought to be physiologically synonymous with neuromodulatory gain control (Moran et al., 2013), in which potassium channels play a central role (Delmas and Brown, 2005). It is possible that the increased expression of *ENGASE* results in the excessive degradation of asparagine-linked N-glycans, thereby altering the functional properties of synaptic potassium channels. If so, then this would inevitably affect neuromodulation, which would be consistent with the loss of gain control of pyramidal cells computationally associated with aberrant sensory precision and reduced MMN (Adams et al., 2013). Deletions of *ENGASE* have been shown in mice to be protective against the embryonic lethality of deletions *NGLY1,* which codes for N-glycanase-1, another deglycosylating enzyme (Fujihira et al., 2017).

The gene set significantly associated with MMN in our analyses at p <0.05 was abnormal neurotransmitter level, indicating that the genes whose expression influences predictive processing in the brain could also be involved in regulating the concentration of neurotransmitters in synaptic clefts. One of the most significantly associated genes within this gene set (shown in the third heatmap in Figure 2) is *KCNJ6,* which encodes the GIRK2 protein, an inward rectifier potassium channel that is ubiquitous in the brain and functionally present in glutamatergic synapses (Saenz del Burgo et al., 2008). The most significant (negatively) associated gene in this gene set was *DLG4*, which encodes PSD95 (postsynaptic density protein 95), another well-studied protein that regulates synapses by trafficking (glutamatergic) NMDA and AMPA receptors (Coley and Gao, 2018). PSD95 is also strongly implicated in schizophrenia and autism (Coley and Gao, 2018). These associations reinforce the notion that MMN and psychosis sharing a genetic component.

We also found that the genes that have more influence over MMN were, overall, underexpressed in adulthood. This could be interpreted to mean that genes that influence MMN (i.e., the genes likely to be involved in establishing the neuronal structures that optimize the short-term plasticity necessary for belief updating) are also involved in early neurodevelopment. This would make sense, as there is a higher likelihood of encountering novel stimuli earlier in life (Köster et al., 2020). The specificity of our neurodevelopmental enrichment results to the frontal cortex is consistent with the source localization of MMN in previous studies (Dima et al., 2012; Ranlund et al., 2016).

Our endophenotype ranking value (ERV) analysis endorses the notion of MMN being an endophenotype of psychosis. The ERV of MMN (0.28) was substantially greater than the ERVs obtained for the other three candidate endophenotypes (0.02–0.13), considering that the maximum possible value in our analysis was 0.447. However, due to the small sample size, the standard errors of the heritability and genetic correlation estimates were large, so this result will require independent replication in a larger sample. ERV is a recent development in the field, so there is limited precedent upon which to specify a minimum sample size for meaningful results. The original article that proposed ERV as a formal approach to the identification of endophenotypes (Glahn et al., 2012) used a sample size of 1,222 individuals to calculate family-based heritability of endophenotypes and their genetic correlation with disease liability. In light of this, there are three factors we consider to be of importance: the heritability of the endophenotype, the heritability of the disease, and the novelty of the findings. The first two are important as the ERV is directly derived from these measures; in this sense, a good sample size for ERV is a good sample size for calculating heritability estimates (Stanton-Geddes et al. [2013] suggest that, with samples drawn from relatively well-controlled environments, sample sizes of a few hundred can yield meaningful SNP-based heritability estimates). Importantly, it has not been possible before to formally assess the utility of MMN as an endophenotype for psychosis, although it is one of the most likely candidates thereof. The ERV we present here for MMN therefore presents a principled starting point for gauging the value of MMN as a psychosis endophenotype.

There are some limitations to the present study. First, as genetic association studies benefit from large samples, independent replication of our research in another large sample would be important. Second, to assemble a large enough dataset for a genetic association study, we combined samples that used slightly different MMN paradigms. These minor differences in methodology were accounted for by combining the samples by meta-analysis, as well as by including testing center as a covariate in the regression analyses. However, future analyses ideally would be carried out in a homogenously tested sample.

In summary, we have laid important groundwork for developing a clearer picture of the neurobiological mechanisms that result in the phenomenon of mismatch negativity and its attenuation in psychosis. Our findings support the use of MMN as an endophenotype for psychosis and implicate *FAM89A* and *ENGASE* as key components of the physiology of prediction error minimization.

## STAR★Methods

### Key Resources Table

**Table:** 

REAGENT or RESOURCE	SOURCE	IDENTIFIER
**Deposited data**		

GTex v7 expression models for Brain Cortex and Frontal Cortex from PredictDB	The University of Chicago	http://predictdb.org/
Developmental Transcriptome RNA-seq data summarized to genes from BrainSpan	BrainSpan Consortium	https://www.brainspan.org/static/download.html
Genome-wide Human SNP Array genotype data (Affymetrix 6.0)	European Bioinformatics Institute, Psychosis Endophenotypes International Consortium	https://www.ebi.ac.uk/ega/dacs/EGAC00001000205

**Software and algorithms**		

PrediXcan	Gamazon et al. (2015)	https://github.com/hakyimlab/PrediXcan
Transcriptome-wide association study gene-set enrichment analysis (TWAS-GSEA)	Pain et al. (2019)	https://github.com/opain/TWAS-GSEA
Newly generated code used to supplement PrediXcan statistical analysis	This paper	https://github.com/abhat92/Transcriptome-wide-association-study-of-mismatch-negativity
lme4qtl (R package)	Ziyatdinov et al. (2018)	https://github.com/variani/lme4qtl
limma (R package)	Ritchie et al. (2015)	https://bioconductor.org/packages/release/bioc/html/limma.html

### Resource availability

#### Lead contact

Requests for further information and resources should be directed to and will be fulfilled by the Lead Contact, Anjali Bhat (anjali.bhat.14@ucl.ac.uk).

#### Materials availability

This study did not generate new unique reagents.

#### Data and code availability

The accession number for microarray data reported in this paper is https://www.ebi.ac.uk/ega/datasets: EGAC00001000205. Previously unpublished code used for statistical analyses reported in this paper is available on GitHub: https://github.com/abhat92/Transcriptome-wide-association-study-of-mismatch-negativity.

### Experimental model and subject details

#### Participants

Participants were drawn from a consortium of three centers: University of Maryland (n = 429), Harvard University (n = 1736) and the London sub-sample of the Psychosis Endophenotypes International Consortium, n = 5635 (Bramon et al., 2014; Ranlund et al., 2016; Shaikh et al., 2012). All samples include patients with psychosis (schizophrenia or bipolar disorder with psychotic symptoms) and healthy controls. MMN data were acquired in a subset of each sample (see Table S1). The London sample additionally contains unaffected relatives of patients with psychosis (*n* = 82). These relatives do not significantly differ in MMN from healthy controls in the same sample (n = 84; see Results), so were treated as healthy controls for the purposes of this study. The collection of data used for this research was approved by the ethics committees at the participating institutions (including King’s College London [References 011/99 and 038/00], the Metropolitan Multi-center Research Ethics Committee [MREC/03/11/090] and University of Maryland). All participants gave written informed consent before they contributed to the study.

#### Clinical assessments

To confirm a DSM-IV or V diagnosis, participants were assessed by a psychiatrist or trained researcher using the following scales: the Positive and Negative Syndrome Scale (Kay et al., 1987), the Schedule for Affective Disorders and Schizophrenia-Lifetime version, for the London and Harvard groups (Endicott and Spitzer, 1978) or the Structured Clinical Interview for DSM-V Axis 1 Disorders, for the Maryland group (First et al., 1997). Family history of any mental disorder was obtained using the Family Interview for Genetic Studies

### Method details

#### MMN data collection and processing

Electroencephalography data were collected using near-identical paradigms at the three centers where participants were recruited (Table S1). Subjects were seated with their eyes open while wearing an electrode cap and presented, through earphones, with sequences of repetitive (standard) auditory stimuli, interspersed with occasional deviant stimuli. To ensure a *pre-attentive* event-related potential was being measured, the subjects were instructed not to pay attention to the sounds presented.

#### Auditory stimulus characteristics

The stimuli presented in the oddball paradigm were 73-80 dB, 1000 Hz tones, with a 0.3 s inter-stimulus interval (from offset to onset of consecutive stimuli). In the Maryland sample (n = 429), 800 tones were presented in one block. In both the London (n = 464) and Harvard (n = 135) samples, 1200 tones were presented in three blocks of 400 tones. The standard stimuli were 60 (Maryland) or 25 (London and Harvard) milliseconds long with a 5ms rise/fall time. These comprised 80% (Maryland) or 85% (London and Harvard) of tones presented. The deviant stimuli were 150ms (Maryland) or 50ms (Harvard and London) long with a 5ms rise/fall time.

#### EEG acquisition

Electroencephalography (EEG) data were collected using arrangements of 21-64 scalp sites (see Table S1 for details of electrode arrangements in each sample) according to the 10/20 International System (all arrangements included the following primary electrodes: FP1, FP2, F7, F8, F3, F4, C3, C4, P3, P4, FZ, CZ, PZ, T3, T4, T5, T6). Recordings were grounded at FPZ using silver/silver-chloride electrodes (Klem et al., 1999) and referenced to the left ear lobe. Eye movements were monitored by vertical, horizontal, and radial electro-oculograms (EoGs). Data were continuously sampled at 1000 Hz (Maryland) or 500 Hz (London and Harvard) with a DC/100 Hz (Maryland) or 0.03 to 120 Hz (Harvard and London) band-pass filter (24 dB/octave roll-off). Impedances were kept below 6 kΩ.

#### EEG pre-processing

Data were re-referenced to common average and band-pass filtered 0.03 (London) or 0.1 (Harvard and Maryland) to 50 Hz. Ocular contamination from the data was removed using the artifact-aligned average procedure (London) (Croft and Barry, 2000) or regression-based weighting coefficients (Harvard and Maryland) (Semlitsch et al., 1986). Data were epoched from 100ms pre-stimulus to 300ms (London) or 400ms (Maryland and Harvard) post-stimulus. Epochs were averaged separately for the standard and deviant tones and then baseline corrected. Mismatch negativity (MMN) was defined as the difference between the deviant and standard event-related potentials. Then the peak MMN (50 to 200ms post-stimulus for Harvard and London; 100 to 250ms post-stimulus for Maryland) was identified by a computer algorithm, which made the process blind to clinical group status. To ensure accurate peak detection, visual inspections of the peaks detected by the algorithm were conducted blind to clinical group and other participant characteristics (Bramon et al., 2004; Hong et al., 2012). This approach (automated detection with blind visual checks) is optimal for large samples and prevents human error and biases.

#### Genetic data collection and processing

DNA was obtained from blood for all participants. We performed genotype imputation separately on each dataset, using information from all individuals that passed genetic quality control, regardless of whether MMN data had been acquired from them. After quality control of the imputed genotypes, 4835, 1602 and 411 individuals and ~6.5, ~7.3 and ~10.1 million SNPs were left for the London, Harvard and Maryland samples, respectively. Of these, EEG data were available for 254, 403 and 71 participants in the London, Maryland and Harvard samples, respectively.

#### Genotyping

The Harvard DNA samples were extracted at the Massachusetts General Hospital Center for Human Genetic Research and genotyped at the Broad Institute using the Illumina OmniExpress Infinium Platform (Illumina Inc., San Diego, CA, USA). The London samples were genotyped with the Genome-wide Human SNP Array 6.0 at the Affymetrix Services Laboratory (https://www.thermofisher.com/us/en/home/life-science/microarray-analysis.html) and sent to the Wellcome Trust Sanger Institute (Cambridge, United Kingdom) for DNA quality control. The Maryland samples were genotyped on the Illumina Omni2.5-8 BeadChip

#### Quality control of genotype data

*London*. Single nucleotide polymorphism (SNP) exclusion criteria for the entire London dataset were: study-wide missing data rate over 5% (11,610 SNPs excluded); having four or more Mendelian inheritance errors identified with PEDSTATS (Wigginton and Abecasis, 2005) (26,585 SNPS excluded); evidence for deviation from Hardy-Weinberg equilibrium (p < 10^−6^; 2,404 SNPS excluded); minor allele frequency < 0.02 (145,097 SNPs excluded); SNPs from X and Y chromosomes or mitochondrial DNA (38,895) and poor genotyping identified by visual inspection of intensity plots in Evoker (Morris et al., 2010)(9499 SNPs excluded).

Sample exclusion criteria for London were: > 2% missing SNP data (214 samples excluded); divergent genome-wide heterozygosity with inbreeding coefficients F > 0.076 or F < −0.076 seen in PLINK (Purcell et al., 2007) (70 samples excluded); chromosomal sharing (inferred from a genome-wide subset of 71,677 SNPs), where 70 duplicates and monozygotic twins were removed by excluding one of each pair (whichever had less complete genotype data) of individuals showing identity by descent > 95%.

*Harvard*. Quality control for the Harvard sample included the following steps: removing individuals with discordant sex information, missing genotype rate > 5% or heterozygosity rate > 3SD, shared IBD > 0.125, or were non-European ancestry based on principal component analyses. Exclusion criteria for SNPs were as follows: SNPs on the X or Y chromosome, MAF < 0.05, call rate < 98%, and p < 1 × 10^−6^ for deviation from Hardy-Weinberg equilibrium. A total of 664,907 autosomal SNPs passed QC. Quality control steps were carried out with PLINK (Purcell et al., 2007).

*Maryland*. Single nucleotide polymorphism (SNP) exclusion criteria for the Maryland dataset were: study-wide missing data rate over 5; evidence for deviation from Hardy-Weinberg equilibrium (p < 1 × 10^−6^) minor allele frequency < 0.01; SNPs from X and Y chromosomes or mitochondrial DNA. Sample exclusion criteria for Maryland were: > 5% missing SNP data (0 samples excluded); divergent genome-wide heterozygosity; identity by descent > 95%. A total of 1799738 autosomal SNPs passed QC. Tables S4,A–D show a full comparison of SNP and sample exclusion criteria across the three datasets.

#### Genotype imputation

Quality controlled genotypes were submitted to the Sanger Imputation Server ((McCarthy et al., 2016) https://imputation.sanger.ac.uk), where the EAGLE2/PWBT (Durbin, 2014; Loh et al., 2016) pipeline was used for pre-phasing and imputation against the Haplotype Reference Consortium panel (r1.1). This yielded ~39.1 million imputed variants. The resulting genotypes were hard-called using a 0.8 genotype probability threshold and all variants with an INFO score < 0.8 were excluded. The original typed genotypes were then merged with the new imputed set such that, where the SNP positions were common to both, the typed data were given preference

#### Quality control of imputed genotypes

QC was performed on imputed genotypes using PLINK. Imputed SNP exclusion criteria were: missing data rate of over 5%; minor allele frequency < 1%; departure from the Hardy-Weinberg equilibrium (p < 1e^-6^); Mendelian error rate > 10%; and cases versus controls data missingness significance < 5e^-6^. Sample exclusion criteria following imputation were: missing data rate of over 5%, Mendelian error rate > 5% and |inbreeding coefficient| > 0.1. LDAK (Speed et al., 2017) was used to identify duplicates or twins as pairs of individuals with a kinship coefficient > 0.95 (based on thinned set of SNPs) and remove one of each pair. After QC, 4835, 1602 and 411 individuals and ~6.5, ~7.3 and ~10.1 million SNPs were left for the London, Harvard and Maryland samples, respectively. Of these, EEG data were available for 254, 403 and 71 participants in the London, Maryland and Harvard samples, respectively. Tables S5, A–C show a full comparison of QC criteria for imputed data across each of the three datasets.

### Quantification and statistical analysis

#### Transcriptome-wide association study (TWAS)

We performed the TWAS using PrediXcan (Gamazon et al., 2015, 2019; Huckins et al., 2019; Wheeler et al., 2016). We obtained SNP-gene expression effect-weights estimated by the PrediXcan developers using GTEx v7 data for brain cortex and frontal cortex from PredictDB (predictdb.org). Entering these expression weights (and the dosage matrices for the corresponding effect alleles) into PrediXcan, we imputed the genetic component of gene expression for 4329 and 3604 genes in the cortex and frontal cortex, respectively. Note that the genes included in the GTEx data only partially overlap between tissues, so although one would expect there to be a high degree of inter-tissue signal sharing, this may not be visible in every instance. We then tested for association between the predicted expression of each gene in each tissue and the amplitude of the MMN at the Fz electrode. As PrediXcan does not allow for the addition of covariates, we entered MMN amplitude values pre-adjusted for clinical group, age, gender and lab where electroencephalographic data were collected. We ran a TWAS separately on each of the three datasets, London (n = 254), Harvard (n = 71) and Maryland (n = 403); and then combined them using a fixed-effect precision-weighted meta-analysis.

#### Gene set enrichment analysis

We performed gene-set enrichment analyses (GSEA) on the cortex and frontal cortex TWAS results for 134 nervous-system function gene-sets (Hall et al., 2020; Pardiñas et al., 2018; Pocklington et al., 2015) from the Mouse Genome Informatics database (Blake et al., 2014). The information we need from this database is, broadly, lists of genes that are known to interact with each other as a part of a ‘pathway’ that performs a particular biological function, in order to assess whether these groups of genes together show significant associations with the phenotype (MMN) in our own sample. It is easier to conduct experiments investigating the functions of gene pathways on mice, which makes this database a rich resource. Furthermore, many such pathways are well conserved through mammalian evolution, so the Mouse Genome Database is commonly used (Bentley et al., 2019; Grama et al., 2020; Hall et al., 2020; Lim and Kim, 2019) as a resource for pathway-based analyses with human samples. Previous human ortholog genome-wide and transcriptome-wide association studies (Grama et al., 2020; Hall et al., 2020) have indeed shown enrichment of gene sets from the Mouse Genome Database.

We used a linear mixed-effects regression-based competitive GSEA approach as previously described (Pain et al., 2019), implemented in TWAS-GSEA (https://github.com/opain/TWAS-GSEA). We used the lme4qtl R package (Ziyatdinov et al., 2018) for mixed model regressions, where the -log_10_ *p-value*s from the TWAS were used as dependent variables. The gene-set membership of each gene was included as a fixed effect predictor and the matrix of correlations between predicted expression of each gene was included as a random effect. The gene correlation matrix was added to the regressions to account for linkage disequilibrium.

#### Neurodevelopmental signature enrichment

We tested for enrichment in our MMN TWAS results of highly expressed or suppressed genes across neurodevelopmental stages. To do this, we downloaded expression data for the two tissues of interest from the BrainSpan Atlas project (www.brainspan.org). The dataset contains RNA-seq data (Reads Per Kilobase of transcript, per Million: RPKMs) for 524 brain tissue samples from 42 individuals (19 females/23 males) aged eight weeks post-conception to 40 years old. RPKM values were log_2_-transformed and lowly expressed genes (log_2_ RPKM < 2^−7^ in 90% or more of the samples; 17389 genes) were subsequently removed. Samples were grouped into nine age-ranges and brain tissue samples were grouped into nine brain regions (Tables S6, A and B). To generate gene expression signatures for each age group (versus the other age groups), we ran linear regressions (Equation 1).

(1) GEx∼β0+β1·AgeGr+β2·AgeGr:CortexRg+β3·BrainRg+β4·Gender+ε

where GEx = gene expression; AgeGr = dummy variable for age-group of interest; CortexRg = dummy variable for region of interest (whole cortex/frontal cortex); BrainRg = variable containing 9 broad brain regions.

Correlation between samples from the same individuals was accounted for by incorporating the intra-donor correlation into the covariance matrix when evaluating regressions. The final age-group expression signatures were generated by fitting a contrast with the sum of the coefficients of the age-group (β1) and of the interaction between age-group and brain region of interest (β2). The differential expression analysis was performed with the R package ‘limma’ (Ritchie et al., 2015). Enrichment for these neurodevelopmental expression signatures in the TWAS results was tested using linear mixed models, using lme4qtl (Ziyatdinov et al., 2018). The logarithm of the *p-value* of each gene in our TWAS (-log_10_ *p-value*) was used as the dependent variable, the signed logarithm of the *p-value*s of the corresponding neurodevelopmental signature (sign (*effect size*) × -log_10_ *p-value*) as the fixed effect predictor and the matrix of correlations between genes as a random effect.

#### Endophenotype ranking of MMN

We also calculated the Endophenotype Ranking Value (ERV) for MMN. The ERV is an index created to objectively quantify the genetic utility of an endophenotype. It varies from 0-1; higher values indicate that the endophenotype and the illness are more strongly influenced by shared genetic factors (Glahn et al., 2012). We specifically calculated the SNP-based ERV of MMN (ERV_SNP_). We used the bivariate Genome-Based Restricted Maximum Likelihood (GREML) function (Lee et al., 2012; Yang et al., 2010) in the Genome-wide Complex Trait Analysis (GCTA) tool (Yang et al., 2011) to estimate the SNP-based heritability of MMN (*h*_e_^2^) in our largest dataset (Maryland; n = 403). Age and gender were included as covariates in this estimation. For schizophrenia (*h*_i_^2^), we extracted the heritability estimate (0.2002), based on a population prevalence of 0.4% (Pardiñas et al., 2018). These two heritability estimates and the genetic correlation (ρ_g_) between MMN and schizophrenia were used to calculate the ERV according to the following equation:

$$ERV_{ie}\;=\;\left|\surd h_{i}^{2}\surd h_{e}^{2}\rho_{g}\right|$$

Note that an ERV of 1 is highly unlikely, as this would mean that the endophenotype and the disorder co-occur with absolute certainty. The maximum possible ERV is also limited by the heritability estimate of the disorder itself. Due to the fixed SNP-based heritability estimate of 0.2002 for schizophrenia the maximum possible ERV would be 0.447 in our analyses. Additionally, the ERV is a ranking value, so it can only be evaluated in comparison to the ERVs of other candidate endophenotypes for the same illness. We calculated the ERVs of three phenotypes in our London sample that have previously shown an association with schizophrenia (Thygesen et al., 2020): lateral ventricular volume, immediate RAVLT (the Ray Auditory Verbal Learning Test, a word recall task) and delayed RAVLT (word-recall after a delay of 30 minutes). These were the three comparator phenotypes (out of 9 initially selected) that had a large enough sample size and low enough standard error to yield meaningful comparisons (Table S7).

## Supplemental information

Document S1. Tables S3–S8 and Figures S1–S3

Table S1. Comparison of the sample sizes, data collection, and processing procedures used in the PEIC, Harvard, and Maryland samples, related to STAR Methods and Tables 1 and 2

Table S2. MMN TWAS results for all genes included in the analysis, related to Figure 1

Document S2. Article plus supplemental information
