# Supplementary material for: Transcriptome-wide association study reveals two genes that influence mismatch negativity
Source: Cell Rep. 2021 Mar 16;34(11):108868. doi: 10.1016/j.celrep.2021.108868 (PMC7972991; doi:10.1016/j.celrep.2021.108868)
Supplement: Document S1. Tables S3–S8 and Figures S1–S3 [file mmc1.pdf]

Cell Reports, Volume *34*

Supplemental information

Transcriptome-wide association study reveals two genes that influence mismatch negativity

Anjali Bhat, Haritz Irizar, Johan Hilge Thygesen, Karoline Kuchenbaecker, Oliver Pain, Rick A. Adams, Eirini Zartaloudi, Jasmine Harju-Seppänen, Isabelle Austin-Zimmerman, Baihan Wang, Rebecca Muir, Ann Summerfelt, Xiaoming Michael Du, Heather Bruce, Patricio O'Donnell, Deepak P. Srivastava, Karl Friston, L. Elliot Hong, Mei-Hua Hall, and Elvira Bramon

Supplemental information

Dataset	Patients vs. controls			
	Intercept	Coefficient	Standard Error	p-value
London	-3.66	0.66	0.22	0.003
Harvard	-4.62	-0.01	0.79	0.99
Maryland	-2.246	0.48	0.17	0.006
Whole sample	-3.84	0.697	0.13	3.46×10^{-08}

Reference group: controls; Effect group: patients

Supplemental Table 3. Results of the linear regressions performed to compare MMN performance between patients and controls. MMN Amplitude (μV) was measured at the Fz electrode. All analyses were adjusted for of age, gender and MMN lab/electroencephalography machine. Related to Table 2.

A

ORIGINAL DATASET					
**London**		**Harvard**		**Maryland**	
**Samples**	**SNPs**	**Samples**	**SNPs**	**Samples**	**SNPs**
5602	929556	1692	719665	429	2335809

B

SAMPLE FILTERS						
	**London**		**Harvard**		**Maryland**	
**Filter**	**Criteria**	**Loss**	**Criteria**	**Loss**	**Criteria**	**Loss**
**Sex mismatch**	remove	57	remove	4	remove	0
**Inbreeding**	<-0.076 / > 0.076	70	<-0.1 / > 0.1	31	<-0.15 / > 0.15	12
**Missingness**	<2%	214	<5%	40	<5%	5
**Duplicates**	0.95 IDB	70	0.95 IDB	15	0.95 IDB	1
**Mendelian errors**	--	--	5%	0	--	--
**Genetic Ancestry**	outlier	356	--	--	--	--

C

SNP FILTERS						
	**London**		**Harvard**		**Maryland**	
**Filter**	**Criteria**	**Loss**	**Criteria**	**Loss**	**Criteria**	**Loss**
**Missingness**	>5%	11610	>5%	17165	>5%	986
**Non-autosomal CHR**	remove	38895	remove	20313	remove	55038
**HWE (p-value)**	$< 1e^{-6}$	2404	$< 1e^{-6}$	14863	$< 1e^{-6}$	26984
**MAF**	<2%	145097	<1%	43442	<1%	462238
**Mendelian errors**	4 errors	26585	10%	100	NA	NA
**Cluster plots**	poor genotyping	9499	--	--	--	--

D

Quality Controlled Dataset					
**London**		**Harvard**		**Maryland**	
**Samples**	**SNPs**	**Samples**	**SNPs**	**Samples**	**SNPs**
4835	691252	1602	627550	411	1799738

Supplemental Tables 4 A-D: Quality control of typed genotypes in each dataset (related to Genetic data collection and processing; STAR Methods).

A

ORIGINAL DATASET					
**London**		**Harvard**		**Maryland**	
**Samples**	**SNPs**	**Samples**	**SNPs**	**Samples**	**SNPs**
4835	39131578	1602	39131578	411	39131578

B

SNP FILTERS						
	**London**		**Harvard**		**Maryland**	
**Filter**	**Criteria**	**Loss**	**Criteria**	**Loss**	**Criteria**	**Loss**
**INFO score**	< 0.8	28048155	< 0.8	20351179	< 0.8	13630162
**> 3 alleles**	remove	9388	remove	15025	remove	29431
**SNP position issues**	remove	787	remove	15102	remove	24105
**Missingness**	> 5%	359398	> 5%	255007	> 5%	145213
**HWE**	$<1e^{-6}$	3803	$<1e^{-6}$	160771	$<1e^{-6}$	111221
**MAF**	< 1%	4246037	< 1%	11070085	<1%	15080028
**Mendelian errors**	10%	21	10%	2404	NA	NA
**Case vs con missingness**	<0.000001	10685	--	--	--	--

C

**Quality Controlled Dataset**

London		Harvard		Maryland	
**Samples**	**SNPs**	**Samples**	**SNPs**	**Samples**	**SNPs**
4835	6454103	1602	7258616	411	10122483

Supplemental tables 5 A-C: Quality control of imputed genotypes in each dataset

Brain structure	Samples	Brain region	Samples	Subjects	Cortex?	Samples	Subjects
anterior (rostral) cingulate (medial prefrontal) cortex	32	cingulate cortex	32	32	yes	361	42
dorsolateral prefrontal cortex	35	frontal cortex	127	38	yes		
orbital frontal cortex	31						
primary motor cortex (area M1, area 4)	26						
ventrolateral prefrontal cortex	35						
occipital neocortex	2	occipital cortex	35	35	yes		
primary visual cortex (striate cortex, area V1/V17)	33						
parietal neocortex	2	parietal cortex	66	35	yes		
posteroventral (inferior) parietal cortex	33						
primary motor-sensory cortex (samples)	5						
primary somatosensory cortex (area S1, areas 3, 1, 2)	26						
inferolateral temporal cortex (area Tev, area 20)	34	temporal cortex	101	40	yes		
posterior (caudal) superior temporal cortex (area 22c)	36						
primary auditory cortex (core)	31						
cerebellar cortex	29	cerebellum	32	32	no	154	40
cerebellum	3						
dorsal thalamus	5	diencephalon	29	29	no		
mediodorsal nucleus of thalamus	24						
amygdaloid complex	33	subcortical	93	37	no		
hippocampus (hippocampal formation)	32						
striatum	28						

Supplemental Table 6 A. Groupings of brain RNASeq samples from the BrainSpan Atlas by brain region. Related to neurodevelopmental enrichment analyses (STAR methods).

<table>
<tr><th>Stage</th><th>Age</th><th>Subjects</th><th>Samples</th><th>Age group</th><th>Subjects</th><th>Samples</th></tr>
<tr><td rowspan="13">PRE-NATAL</td><td>8 pcw
9 pcw
12 pcw</td><td>1
1
3</td><td>12
9
45</td><td>8 - 12 pcw</td><td>5</td><td>66</td></tr>
<tr><td>13 pcw
16 pcw</td><td>3
3</td><td>44
39</td><td>13 - 16 pcw</td><td>6</td><td>83</td></tr>
<tr><td>17 pcw
19 pcw
21 pcw
24 pcw</td><td>1
1
2
1</td><td>14
11
16
16</td><td>17 - 24 pcw</td><td>5</td><td>57</td></tr>
<tr><td>25 pcw
26 pcw
35 pcw
37 pcw</td><td>1
1
1
1</td><td>1
3
2
16</td><td>25 - 37 pcw</td><td>4</td><td>22</td></tr>
<tr><td rowspan="5">POST-NATAL</td><td>4 mos
10 mos
1 yrs</td><td>3
1
1</td><td>33
10
16</td><td>4 mos - 1 yr</td><td>5</td><td>59</td></tr>
<tr><td>2 yrs
3 yrs
4 yrs</td><td>1
2
1</td><td>12
25
7</td><td>2 - 4 yrs</td><td>4</td><td>44</td></tr>
<tr><td>8 yrs
11 yrs
13 yrs
15 yrs</td><td>2
1
1
1</td><td>27
14
16
5</td><td>8 - 15 yrs</td><td>5</td><td>62</td></tr>
<tr><td>18 yrs
19 yrs
21 yrs
23 yrs</td><td>1
1
1
1</td><td>13
16
16
14</td><td>18 - 23 yrs</td><td>4</td><td>59</td></tr>
<tr><td>30 yrs
36 yrs
37 yrs
40 yrs</td><td>1
1
1
1</td><td>16
16
16
15</td><td>30 - 40 yrs</td><td>4</td><td>63</td></tr>
</table>

pcw = post-conception weeks; mos = months; yrs = years

Supplemental Table 6B. Groupings of brain RNASeq samples from the BrainSpan Atlas by age. Related to neurodevelopmental enrichment analyses (STAR Methods).

Phenotype	N	GCTA possible	Error	ERV	Heritability			Genetic correlation		
					Estimate	S.E.[a]	95% C.I.	rG	S.E.[a]	95% C.I.[b]
Digit Symbol	27	no	Variance-covariance matrix invertibility	NA	NA	NA	NA	NA	NA	NA
Digit Span Forward	72	yes	NA	0.41	0.83	0.37	[0.10 ; 1.56]	-1.00	1.43	[-3.80 ; 1.80]
IQ	324	no	Likelihood convergence	NA	NA	NA	NA	NA	NA	NA
Mismatch negativity	403	yes	NA	0.28	0.38	0.15	[0.09 ; 0.68]	1.00	0.22	[0.58 ; 1.42]
P300 Amplitude	510	yes	NA	0.36	0.64	0.17	[0.31 ; 0.97]	1.00	4.55	[-7.93 ; 9.93]
P300 Latency	515	no	Variance-covariance matrix invertibility	NA	NA	NA	NA	NA	NA	NA
Lat. ventricular volume	775	yes	NA	0.02	0.66	0.17	[0.35 ; 0.97]	-0.05	0.27	[-0.58 ; 0.48]
Whole brain volume	777	no	Likelihood convergence	NA	NA	NA	NA	NA	NA	NA
RAVLT delayed	2384	yes	NA	0.10	0.34	0.06	[0.22 ; 0.46]	-0.39	0.10	[-0.59 ; -0.19]
RAVLT immediate	2406	yes	NA	0.13	0.35	0.06	[0.23 ; 0.48]	-0.50	0.10	[-0.68 ; -0.31]
Block Design	3089	yes	NA	0.34	0.69	0.05	[0.59 ; 0.79]	-0.93	5.14	[-11.01 ; 9.15]

[a] S.E = Standard error

[b] C.I. = Confidence intervals

ERV = Endophenotye Ranking Value

RAVLT = Ray Auditory Verbal Learning Task

Supplemental Table 7: Results of the bivariate GREML analyses performed with GCTA. Related to endophenotype ranking analysis (STAR Methods).

Sample	Patients	Controls	Relatives	Whole dataset
**Maryland (*n* = 403)**	190.9 ± 27.22[a]	180.33 ± 31.88	-	186.6 ± 29.63
**Harvard (*n* = 66)**	174.38 ± 33.07	198.36 ± 25.97	-	179.83 ± 33.0
**PEIC (*n* = 100)**	98.58 ± 19.69	100.54 ± 16.61	103.08 ± 14.67	100.75 ± 17.09

[a]Mean ± Standard deviation (in ms);

[b]These values are unadjusted for covariates;

Supplemental Table 8. Mean mismatch negativity latency[b] at FZ (ms) in each of the datasets by group. Related to Table 2.

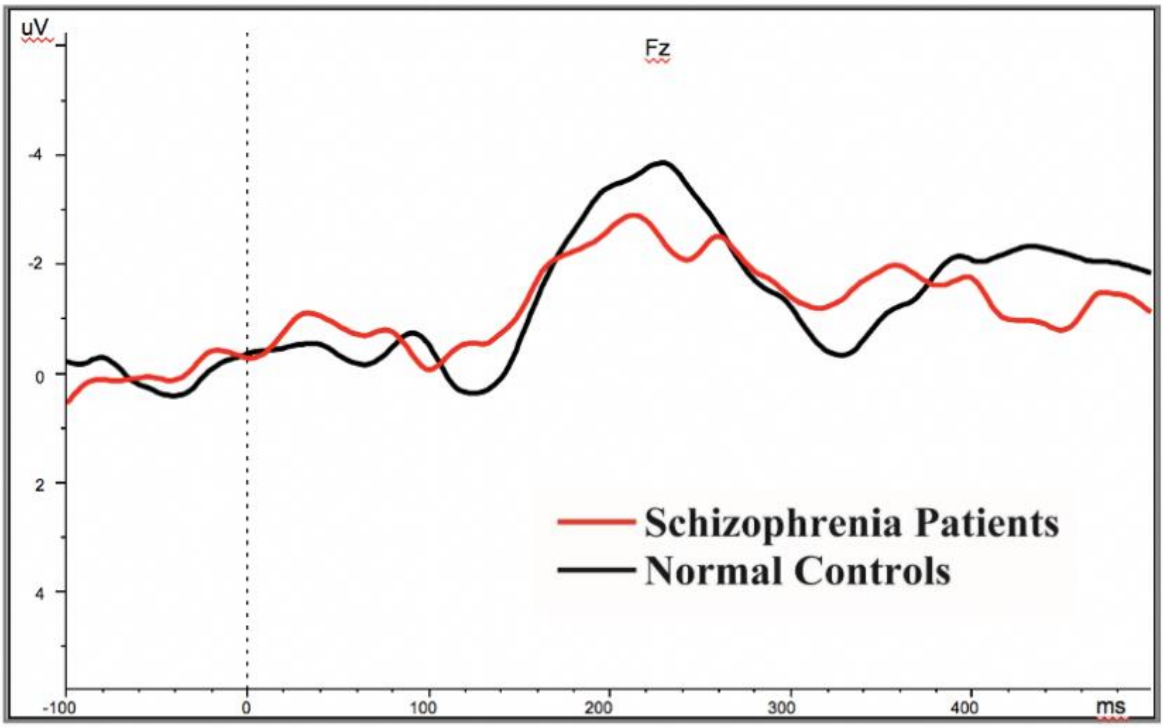

Supplemental Figure 1. MMN FZ grand average waveforms for patients (red) and controls (black) in the Harvard sample (*n* = 71). Related to Table 2.

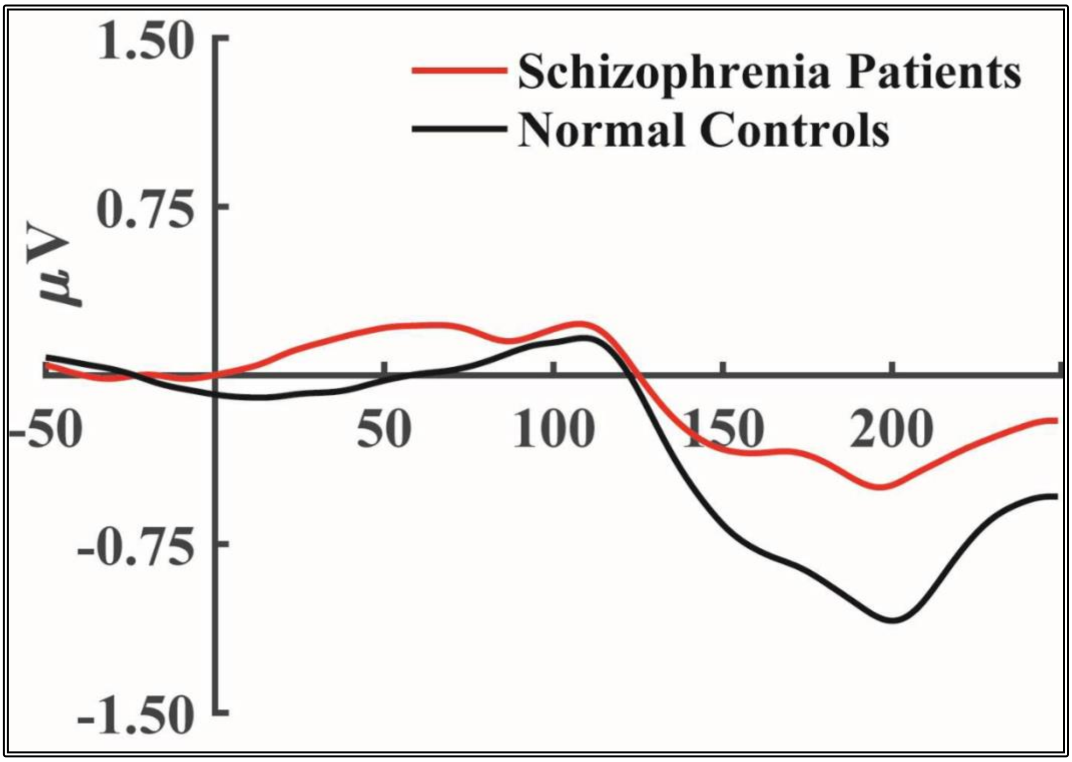

Supplemental Figure 2. MMN FZ grand average waveforms for patients (red) and controls (black) in the Maryland sample (*n* = 403). Related to Table 2.

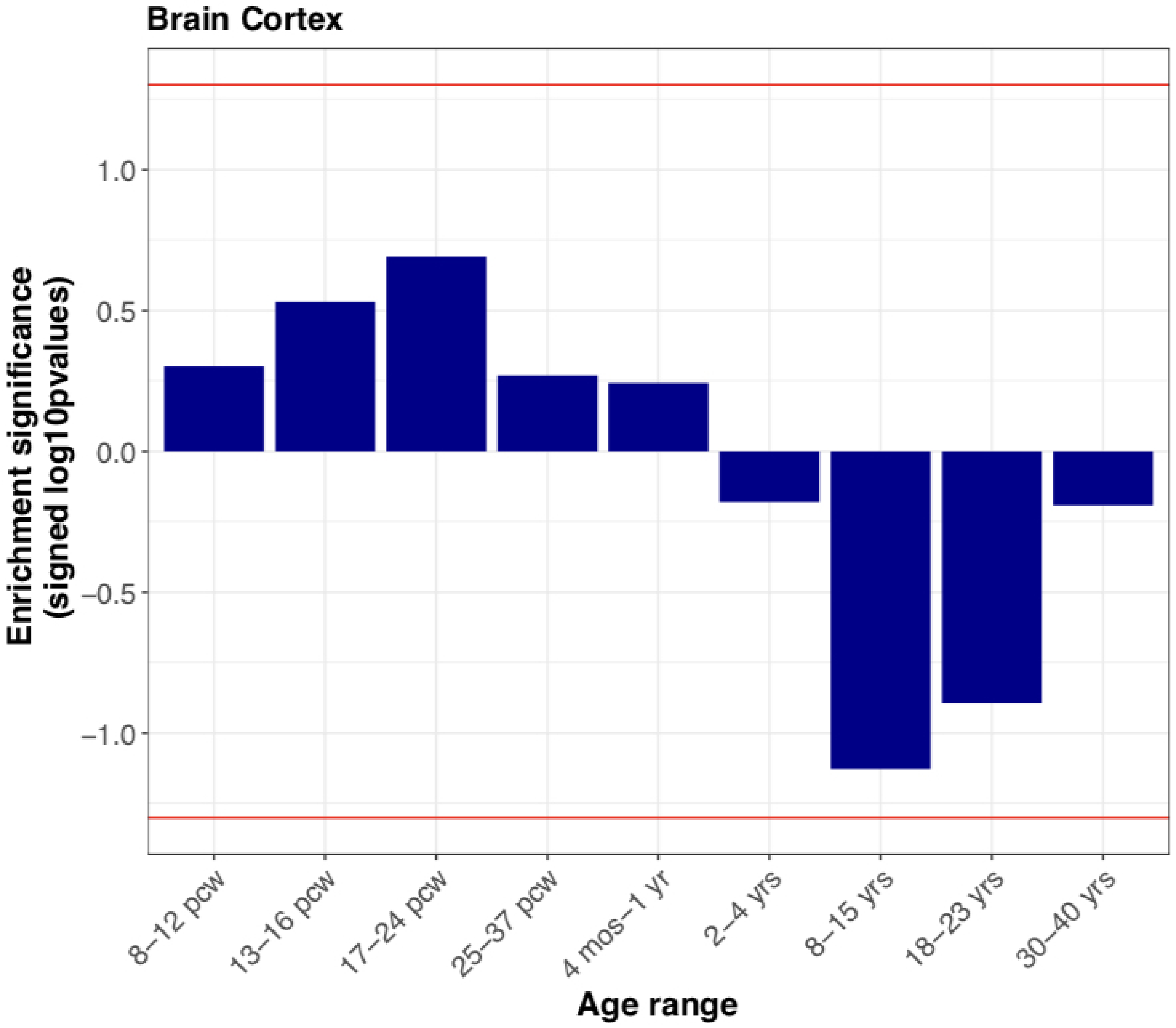

Supplemental Figure 3. Neurodevelopmental signature enrichment results for whole cortex. Related to Figure 3.
